# A Novel Tetramethylpyrazine Derivative Prophylactically Protects against Glutamate-Induced Excitotoxicity in Primary Neurons through the Blockage of *N*-Methyl-D-aspartate Receptor

**DOI:** 10.3389/fphar.2018.00073

**Published:** 2018-02-12

**Authors:** Shengquan Hu, Huihui Hu, Shinghung Mak, Guozhen Cui, Mingyuen Lee, Luchen Shan, Yuqiang Wang, Huangquan Lin, Zaijun Zhang, Yifan Han

**Affiliations:** ^1^Institute of New Drug Research and Guangzhou Key Laboratory of Innovative Chemical Drug Research in Cardio-cerebrovascular Diseases, Jinan University College of Pharmacy, Guangzhou, China; ^2^Department of Applied Biology and Chemical Technology, Institute of Modern Chinese Medicine, The Hong Kong Polytechnic University, Hong Kong, China; ^3^Shenzhen Research Institute, The Hong Kong Polytechnic University, Shenzhen, China; ^4^Department of Bioengineering, Zunyi Medical University, Zhuhai, China; ^5^State Key Laboratory of Quality Research in Chinese Medicine and Institute of Chinese Medical Sciences, University of Macau, Taipa, Macau; ^6^Shenzhen Research Institute, The Chinese University of Hong Kong, Shenzhen, China

**Keywords:** Alzheimer’s disease, tetramethylpyrazine derivative, NMDA receptor, neuroprotection, oxidative stress, GSK3β

## Abstract

The over-activation of NMDA receptor via the excessive glutamate is believed to one of the most causal factors associated with Alzheimer’s disease (AD), a progressive neurodegenerative brain disorder. Molecules that could protect against glutamate-induced neurotoxicity may hold therapeutic values for treating AD. Herein, the neuroprotective mechanisms of dimeric DT-010, a novel derivative of naturally occurring danshensu and tetramethylpyrazine, were investigated using primary rat cerebellar granule neurons (CGNs) and hippocampal neurons. It was found that DT-010 (3–30 μM) markedly prevented excitotoxicity of CGNs caused by glutamate, as evidenced by the promotion of neuronal viability as well as the reversal of abnormal morphological changes. While its parent molecules did not show any protective effects even when their concentration reached 50 μM. Additionally, DT-010 almost fully blocked intracellular accumulation of reactive oxygen species caused by glutamate and exogenous oxidative stimulus. Moreover, Western blot results demonstrated that DT-010 remarkably attenuated the inhibition of pro-survival PI3K/Akt/GSK3β pathway caused by glutamate. Ca^2+^ imaging with Fluo-4 fluorescence analysis further revealed that DT-010 greatly declined glutamate-induced increase in intracellular Ca^2+^. Most importantly, with the use of whole-cell patch clamp electrophysiology, DT-010 directly inhibited NMDA-activated whole-cell currents in primary hippocampal neurons. Molecular docking simulation analysis further revealed a possible binding mode that inhibited NMDA receptor at the ion channel, showing that DT-010 favorably binds to Asn602 of NMDA receptor via arene hydrogen bond. These results suggest that DT-010 could be served as a novel NMDA receptor antagonist and protect against glutamate-induced excitotoxicity from blocking the upstream NMDA receptors to the subsequent Ca^2+^ influx and to the downstream GSK3β cascade.

## Introduction

Alzheimer’s disease is a devastating progressive brain disorder that severely destroys the learning and memory function of patients. It is estimated that nearly 44 million people develop AD and other related dementia. AD is primarily characterized by progressive loss of functional neurons mainly located in the hippocampal and cortical brain regions. Although the pathogenesis of AD is complicated and multifaceted, it is generally accepted that glutamate excitotoxicity contributes severely to the neuronal degeneration observed in AD (Paoletti et al., 2013; Wang and Reddy, 2017).

Substantial evidence supports that glutamate is the most important excitatory neurotransmitter in mammals regulating synaptic plasticity (Colbran, 2015). The NMDA receptor is a glutamate receptor and ion channel protein mainly existing in neurons. These receptors gate the cytoplasmic influx of Ca^2+^, depending on the intensity of the stimulus, and subserve either physiological functions or neuronal death (Contestabile, 2012; Korde and Maragos, 2012). Under normal circumstances, physiological activation of NMDA receptor at synapses induces synaptic plasticity, a cellular mechanism for memory formation. While under pathological states, over-activation of NMDA receptors by abnormal release of glutamate has been demonstrated to mediate excitotoxic neuronal damage observed in AD and other neuropathological conditions (Luo et al., 2010). More specifically, excessive glutamate pathologically over-activates NMDA receptors in the post-synaptic neurons, then causes a massive abnormal influx of extracellular Ca^2+^ into cells and finally leads to neuronal death. Several possible signaling pathways, including oxidative stress, activation of mitogen-activated protein kinase and nitric oxide synthase, as well as inhibition of PI3-K/Akt/GSK3β have been proposed for Ca^2+^- triggered downstream cascades (Hardingham, 2009; Ghosh et al., 2012; Baek et al., 2017; Dar et al., 2017). At the late stage of excitotoxicity, glutamate initiates mitochondria-dependent apoptotic cascade by altering the expression of apoptosis regulators such as Bax, Bcl-2, Bid (Zhang and Bhavnani, 2006; Chen et al., 2012). A shift in the balance of these proteins in turn activates caspase-3 and disrupts mitochondrial membrane potential, and finally causes extensive neuronal death.

The hippocampus is a critical region of brain that plays important roles in learning and memory, and one of the first components of the brain to suffer damage in AD. It has been well documented that hippocampal neurons are the most sensitive effector to various neurotoxins particularly including β-amyloid, 1-methyl-4-phenylpyridinium, H_2_O_2_, NMDA and glutamate (Luo et al., 2010; Wang Y. et al., 2017). More importantly, evidence has indicated that whole-cell patch clamp electrophysiology of hippocampal neurons represents the most practical approach for high-fidelity investigation of underlying biophysical mechanisms (Kodandaramaiah et al., 2012).

Similarly, primary CGNs that well express NMDA receptors are widely used in the field of neuroscience and neuropharmacology (Contestabile, 2002; Xifro and Rodriguez-Alvarez, 2015). These cells are popular because it is easy, *in vitro*, to acquire abundant and homogeneous CGNs (over 95% are CGNs in the cerebellum) from the explanted cerebellum than other neurons (hippocampal and cortical neurons). More encouragingly, stimulus particularly including glutamate initiate the similar signaling cascades in CGNs as those in the hippocampal and cortical neurons (Contestabile, 2002). Increasing lines of evidence indicate that the glutamate excitotoxic CGNs model represent a very practical model system for evaluating the neuroprotective ability of drugs against neurodegenerative disorders, particularly AD (Wang et al., 2014; Hu et al., 2015a).

**FIGURE 1:**
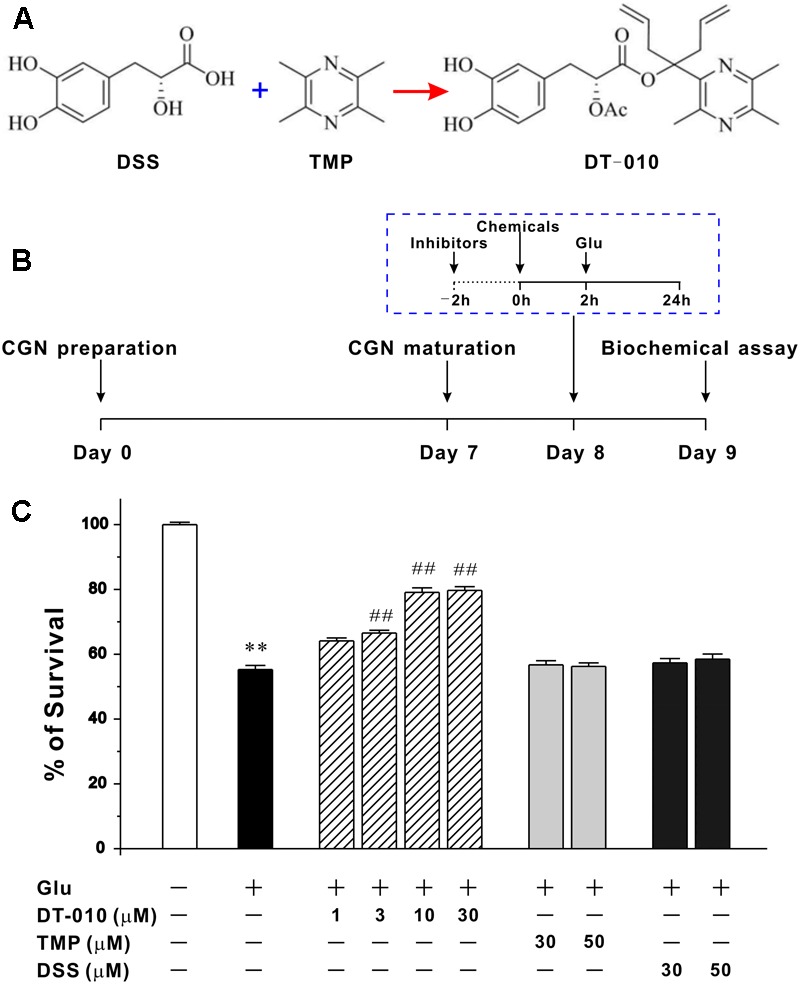
DT-010, but not TMP and DSS, concentration-dependently protects against glutamate-induced excitotoxicity in primary CGNs. **(A)** Chemical structures of DSS, TMP and DT-010. **(B)** Schematic diagram demonstrates the timeline and general procedures for examining the neuroprotective effects of tested chemicals. At 8 DIV, CGNs were pre-treated with or without tested chemicals for 2 h, then exposed to 100 μM glutamate (Glu), and finally subjected to various biochemical assays after glutamate insult. **(C)** DT-010 concentration-dependently prevents excitotoxicity in CGNs caused by glutamate. CGNs were pre-treated with DT-010 (1–30 μM), TMP (30–50 μM) or DSS (30–50 μM) for 2 h, then exposed to 100 μM glutamate for 24 h. Neuronal viability was measured by MTT method ^∗∗^*p* < 0.01, *vs.* control group; #*p* < 0.05, ##*p* < 0.01, *vs.* glutamate group.

The rhizome of *Salvia miltiorrhiza* Bge. and *Ligusticum chuanxiong* Hort. are traditional Chinese medicinal herbs and widely used in China for a long time. Danshensu (3-(3, 4-dihydroxy-phenyl) lactic acid, DSS) and TMP are the effective components isolated from *Salvia miltiorrhiza* Bge. and *Ligusticum chuanxiong* Hort. respectively. Previous studies indicated that these components possess a variety of pharmacological activities, including cardioprotection (Wang C. et al., 2017), anti-inflammation (Yin et al., 2011), and antimyocardial ischemic damage (Yin et al., 2013). Structural combination of two ingredients with known pharmacological properties may generate a novel compound embracing multiple and unique properties (Chen et al., 2015). We previously developed and synthesized a hetero-dimer (DT-010) in which TMP was linked to DSS via an ester bond and two allyl groups at the carboxyl group (**Figure 1A**) (Cui et al., 2014). More recently, we have demonstrated that DT-010 was able to enhance the chemotherapeutic efficacy of doxorubicin in tumor cells (Wang et al., 2016). Additionally, DT-010 could offer cardioprotection and neuroprotection against Parkinson’s disease-related neurotoxins (Hu et al., 2016; Zhang et al., 2017). However, the neuroprotective effects of DT-010 in cellular models associated with AD have not been investigated. Herein, we explored the neuroprotective mechanisms by which DT-010 protected against glutamate-induced neurotoxicity in rat primary CGNs and hippocampal neurons using various biochemical and physiological assays.

## Materials and Methods

### Reagents

3(4,5-dimethylthiazol-2-yl)-2.5-diphenyltetrazolium bromide, LY294002, Hoechst 33342, SB415286, DCFH-DA, FDA, PI and L-glutamic acid hydrochloride (glutamate) were from Sigma Chemicals (St. Louis, MO, United States). Cell culture medium and fetal bovine serum were from Gibco (Carlsbad, CA, United States). Phospho-Ser473-Akt, total Akt, phospho-Ser9-GSK3β, total GSK3β and β-actin antibodies were from Cell Signaling Technology (Beverly, MA, United States).

### Preparation of Primary Rat CGNs

All animal studies were approved by the Committee on the Use of Animals in Teaching or Research (No. 16-17_38-ABCT-R-GRF) at the Hong Kong Polytechnic University. All animals were purchased from the Centralized Animal Facilities (CAF) at the Hong Kong Polytechnic University. Rat CGNs were obtained from 8-day-old SD rats as described by us (Hu et al., 2013). Briefly, the dissected cerebella from early postnatal rat were digested with 0.25% trypsin for 15 min and then triturated to obtain a single cell suspension. CGNs were used after 8 days *in vitro* (8 DIV).

### Preparation of Primary Rat Hippocampal Neurons

Rat hippocampal neurons were prepared from 18-day-old SD rat embryos as reported by us (Luo et al., 2010). In brief, the dissected hippocampi were digested and dissociated using a Pasteur pipette. These neurons were seeded at a density of 2.0 × 10^5^ cells/ml in neurobasal/B27 medium supplemented with 500 μM glutamine, 10% FBS, 100 units/ml penicillin and 100 μg/ml streptomycin. Hippocampal neurons were used for patch clamp electrophysiology after 15 days *in vitro*.

### Drug Treatment of CGNs

Cerebellar granule neurons were pre-treated for 2 h with increasing concentration of tested chemicals (DT-010, TMP, DSS, SB415286), then incubated with glutamate (100 μM) for 24 h (cell viability measurement) or 6 h (phosphorylated protein level examination). For the study of molecular pathways involved, inhibitors were used 2 h prior to the administration of tested chemicals.

### MTT Assay

Neuronal viability was carried out using MTT reduction assay as reported (Hu et al., 2015a). After a 24 h of glutamate insult, cells in 96-well plates were treated with 10 μl of MTT solution (stock: 5 mg/ml) and further incubated for 4 h. The absorbance was measured at a 570 nm wavelength.

### FDA/PI Double Staining

The measurement of live/dead neurons were carried out using FDA/PI staining as reported (Hu et al., 2013). After treatment, CGNs were stained simultaneously with FDA (10 μg/ml) and PI (5 μg/ml) for 5 min, then photographed using the fluorescence microscope (Nikon Instruments Inc., Melville, NY, United States).

### Hoechst 33342 Staining

The evaluation of apoptotic chromatin condensation was performed using Hoechst staining as we previously described with minor modification (Hu et al., 2013). Twenty four hour after glutamate insult, CGNs were stained with Hoechst 33342 (5 μg/ml) for 5 min, and the apoptotic nuclei were photographed using the microscope.

### Assay of Intracellular Accumulation of Reactive Oxygen Species (ROS)

Reactive oxygen species generation was measured by using the redox-sensitive fluorescent probe DCFH-DA as reported (Hu et al., 2015b). Briefly, 24 h after glutamate insult, CGNs were incubated for 15 min with DCF-DA (10 μM) in the dark. Fluorescence was recorded by a microplate reader with an excitation wavelength of 495 nm and an emission wavelength of 515 nm.

### Determination of DPPH Radicals Scavenging Activities

DPPH radical is a widely used method to evaluate the free radical scavenging ability of natural compounds. Appropriate dilutions of DT-010 (100 μl) were mixed with 100, 50 μM methanolic solution containing 2,2-diphenyl-1-picrylhydrazyl radicals (DPPH∙), the mixtures were left in the dark for 50 min at room temperature and the absorbance was measured at 517 nm using a plate reader (BioTek Synergy 5, Winooski, VT, United States). The clearance of the DPPH∙ radical was calculated as follows: DPPH Clearance (%) = [(A_ctrl_–A_sample_) /A_ctrl_] × 100, where A_ctrl_ and A_sample_ were defined as the absorbance of the control and the samples.

### Confocal Ca^2+^ Imaging

The concentration of intracellular Ca^2+^ was monitored by observing the fluorescence of Fluo-4 probe using Leica TCS SPE Confocal microscope (Leica Microsystems, Wetzlar, Germany) with the Leica LAS X software. In short, CGNs were incubated with Fluo-4 AM (1 μM) for 30 min, and then washed twice to remove extracellular Fluo-4 AM. Intracellular Ca^2+^ image with green fluorescence was taken when CGNs were treated with glutamate in the presence or absence of DT-010. Data were acquired by measuring the fluorescence (*F*) from selected areas within a neuron, following the subtraction of the background fluorescence, and division by the fluorescence intensity before drug administration (*F*_0_), expressed as *F*/*F*_0_. DT-010 (30 μM) was introduced into the system 2 h prior to glutamate (100 μM).

### Whole-Cell Patch Clamp Electrophysiology

Whole-cell patch clamp technique was performed in primary hippocampal neurons using an Axopatch 200B patch amplifier as we previously described (Luo et al., 2010). Briefly, matured neurons were constantly superfused with solutions buffered (in mM) by 0.25 CaCl_2_, 150 NaCl, 10 glucose, 5 KCl, 0.001 glycine, 10 tetrodotoxin, 0.01 (-)-bicuculline methiodide and 10 HEPES. A low calcium level were introduced to decrease the calcium-dependent desensitization of NMDA-evoked currents. Pipettes pulled from borosilicate glass had a resistance of 2–4 MΩ when filled with a pipette solution that contained 140 mM CsCl, 10 mM EGTA, 10 mM HEPES and 5 mM MgATP (pH, 7.3) and 315 mM mOsm in osmolarity.

### Western Blotting Analysis

The signaling pathways involved were probed using Western blotting as reported by us (Hu et al., 2015a). CGNs were lysed on ice and scraped from the 6-well plates. The cellular protein was then collected by centrifugation (12,000 rpm, 10 min, 4°C) and quantified using bicinchonnic acid assay. 30–60 μg of proteins were then separated in SDS-PAGE gel. After the transfer of proteins to PVDF membranes, first (diluted in TBST containing 0.5% bovine serum albumin, 1:1000 for phospho-Ser473-Akt, total Akt, phospho-Ser9-GSK3β, and total GSK3β, 1: 2000 for β-actin) and secondary antibodies (1:2000 dilution) were added, respectively. The blots were then developed and exposed to *x*-ray films.

### Molecular Docking Simulation

Molecular docking was carried out using the default parameters of MOE software (version 2016. 08, Chemical Computing Group Inc., Montreal, QC, Canada). The molecular model of NMDA receptor was built from the X-ray co-crystal structure of NMDA receptor (PDB: 5UOW) in complex with one ligand, the ion channel blocker (MK-801). The crystallographic structure was prepared using QuickPrep program of MOE using default setting. DT010 was organized in MOE database file. The co-crystallized ligand MK-801 for the identification for the binding site.

### Statistical Analysis

All the data were acquired from at least 3 independent experiments and presented as means ± S.E.M. The statistical significance was evaluated by One-way analysis of variance. *p* < 0.05 was considered to be significant.

## Results

### DT-010 Concentration-Dependently Prevents Glutamate-Induced Excitotoxicity in Primary CGNs

We had successfully established the cellular model in which the neurotoxicity is triggered by glutamate in primary CGNs (Hu et al., 2013). The timeline and general procedures for examining the neuroprotective effects of tested chemicals using this model was shown in **Figure 1B**. Twenty four hour exposure of CGNs to 100 M glutamate caused a (55.2 ± 1.3) % loss of neurons. While 2 h pre-treatment with DT-010 (3, 10, and 30 μM) markedly prevented glutamate-induced excitotoxicity, promoting neuronal viability to (66.5 ± 0.8) %, (74.5 ± 1.3) % and (79.7 ± 1.2) %, respectively (**Figure 1C**). In contrast, its parent molecules, TMP and DSS, failed to protect CGNs even when their concentrations was up to 50 μM (**Figure 1C**). And notably, in a co- and post-treatment experimental paradigm, DT-010 could not exhibit significant restorative effects against glutamate-induced CGNs damage (data not shown).

### DT-010 Greatly Attenuates the Morphological Abnormal Changes of CGNs Caused by Glutamate

**FIGURE 2:**
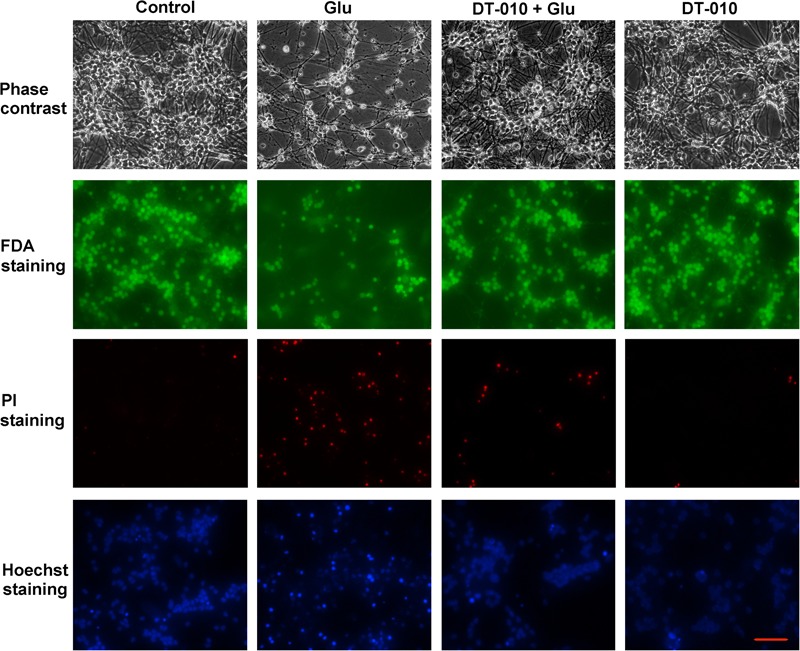
DT-010 effectively attenuates the morphological abnormal changes caused by glutamate in CGNs. CGNs were pre-incubated in the presence or absence of DT-010 (30 μM) for 2 h, then exposed to 100 μM glutamate for 24 h, and stained with FDA/PI or Hoechst 33342, observed using phase-contrast and Nikon fluorescence microscopes.

To further confirm the neuroprotective effects of DT-010, the morphological changes of CGNs treated with or without DT-010 (30 μM) in the presence of glutamate were observed. As demonstrated in **Figure 2**, phase contrast microscopy images indicated that DT-010 effectively inhibited the morphological abnormal changes caused by glutamate (100 μM, 24 h), including unhealthy cell bodies and broken neuritic networks. In addition, 24 h exposure of CGNs to glutamate resulted in a significant decrease in FDA-stained viable neurons and increase in PI-positive dead cells, DT-010 greatly reversed these changes. Furthermore, blue fluorescent Hoechst 33342 brightly stained the condensed chromatin of apoptotic neurons in glutamate group, but more dimly stained the normal chromatin of live neurons in groups of control and DT-010 plus glutamate.

### DT-010 Almost Fully Blocks the Intracellular Accumulation of ROS and Protects CGNs against Exogenous Oxidative Stimuli

**FIGURE 3:**
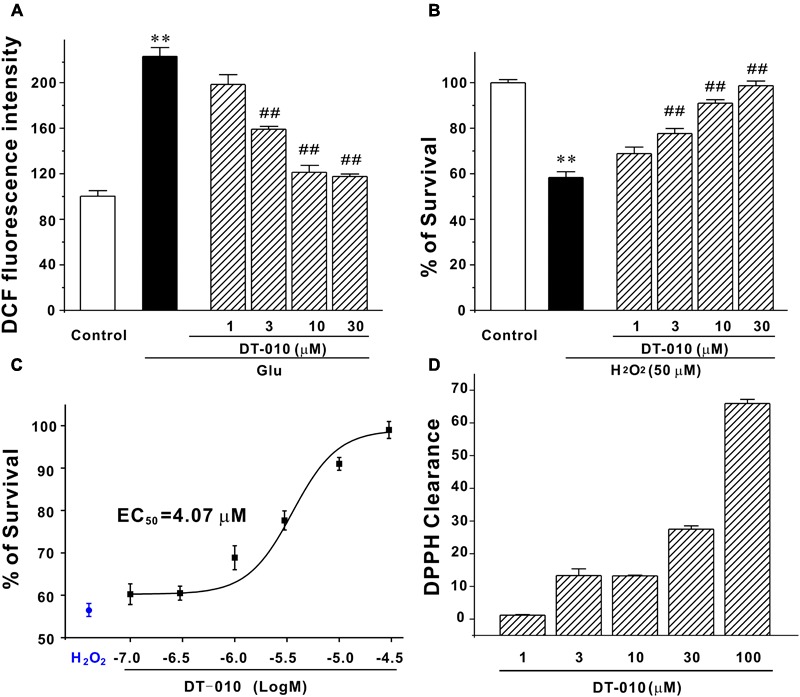
DT-010 fully blocks endogenous and exogenous oxidative damage in CGNs. **(A)** DT-010 almost fully blocks the intracellular accumulation of ROS caused by glutamate. CGNs were pre-treated with or without DT-010 (1–30 μM) for 2 h, followed by exposure to 100 μM glutamate for another 24 h. Intracellular accumulation of ROS was measured by DCFH-DA probe. ^∗∗^*p* < 0.01, compared to control group; ##*p* < 0.01, *vs.* glutamate group. **(B)** DT-010 completely protects against exogenous oxidative stress. CGNs were pre-treated with or without DT-010 (1–30 μM) for 2 h, then incubated with 50 μM H_2_O_2_ for 6 h, and subjected to MTT assay. ^∗∗^*p* < 0.01, *vs.* control group; ##*p* < 0.01, *vs.* H_2_O_2_ group. **(C)** DT-010 provides neuroprotective effects against H_2_O_2_–induced cytotoxicity with an EC_50_ value of 4.07 μM. CGNs were treated as in **(B)** and the EC_50_ value was calculated using Origin Pro 8 statistical software. **(D)** DT-010 exhibits significant strong DPPH radical scavenging activity.

It has been reported that oxidative damage contributes severely to the glutamate-induced excitotoxicity (Akanda et al., 2017), we then examined the possibility that DT-010 may protect against endogenous accumulation of ROS caused by glutamate. As evident in **Figure 3A**, DT-010 greatly attenuated ROS accumulation caused by glutamate (100 μM, 24 h). Particularly, DT-010 at 30 μM almost fully blocked ROS generated in CGNs. Moreover, to examine whether DT-010 could directly prevent exogenous oxidative stimuli, we introduced a popular cellular model in which neurotoxicity was triggered by H_2_O_2_ in CGNs. Similarly, DT-010 completely protected CGNs from H_2_O_2_-induced toxicity, with an EC_50_ value of 4.07 μM (**Figures 3B,C**). Most encouragingly, the direct anti-oxidative effects of DT-010 were further investigated using DPPH radicals scavenging assay, a method for evaluating the free radical scavenging ability of chemicals. It was found that DT-010 exhibited significant strong DPPH radical scavenging activity ranging from 3 to 100 μM, suggesting that DT-010 may act as a direct antioxidant (**Figure 3D**).

### DT-010 Activates PI3K/Akt/GSK3β Pathway in CGNs

**FIGURE 4:**
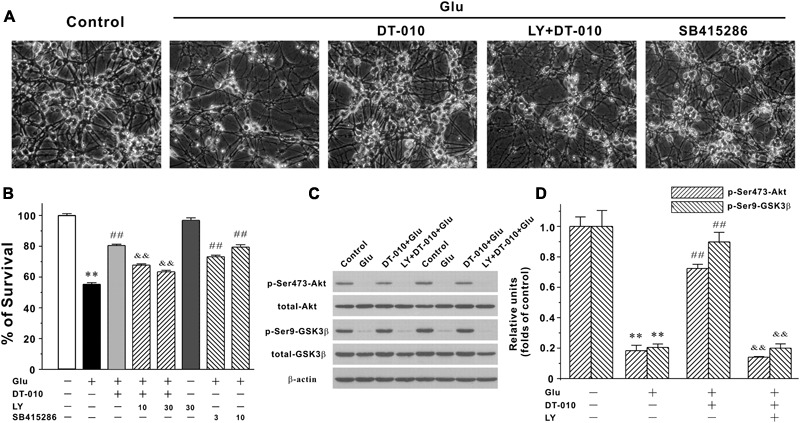
DT-010 effectively reverses the inhibition of PI3-K/Akt/GSK3β pathway caused by glutamate in CGNs. **(A,B)** PI3-K inhibitor significantly abolishes the neuroprotective effects of DT-010. CGNs were pre-treated with or without LY294002 (LY, PI3-K inhibitor) for 2 h, then incubated with DT-010 (30 μM) for another 2 h, followed by 24 h exposure of glutamate. For testing the possibility that GSK3β pathway was involved in the glutamate-induced excitotoxicity, CGNs were pre-treated with SB415286 (GSK3β inhibitor) for 2 h, followed by 24 h incubation of glutamate. Thereafter, CGNs were observed using the phase-contrast microscope **(A)** and subjected to MTT assay for measuring neuronal viability **(B)**, respectively. ^∗∗^*p* < 0.01, *vs.* control group; ##*p* < 0.01, *vs.* glutamate group; &&*p* < 0.01, *vs.* DT-010 plus glutamate group. **(C,D)** PI3-K inhibitor blocks the DT-010-mediated reversal of phosphorylated Akt and GSK3β. CGNs were pre-treated with or without LY294002 (LY, PI3-K inhibitor) for 2 h, then incubated with DT-010 for another 2 h, followed by 6 h exposure of glutamate. After that, CGNs were harvested and the extracted protein was subjected to Western blotting analysis. ^∗∗^*p* < 0.01, *vs.* control group; ##*p* < 0.01, *vs.* glutamate group; &&*p* < 0.01, *vs.* DT-010 plus glutamate group.

Since the inhibition of pro-survival PI3K/Akt/GSK3β pathway plays a critical role in the neurodegeneration caused by glutamate (Dar et al., 2017), it is reasonable to ask whether DT-010 may provide its neuroprotection through activating PI3K/Akt/GSK3β pathway. First, SB415286 (3 and 10 μM, 2 h pre-treatment), the specific inhibitor of GSK3β, effectively protected CGNs by inhibiting the morphological changes and enhancing neuronal viability (**Figures 4A,B**), indicating the involvement of GSK3β pathway in neurodegeneration caused by glutamate (100 μM, 24 h). Second, LY294002 (LY, 10 and 30 μM, 2 h pre-treatment), the specific blocker of PI3K, abrogated DT-010-mediated neuroprotection (**Figures 4A,B**). Moreover, we had previously established the time course of protein levels of p-Ser473-Akt and p-Ser9-GSK3β in response to glutamate insult (Hu et al., 2013), and found that phosphorylated Akt and GSK3β dramatically decreased after 6 h exposure of glutamate. In this study, we therefore investigated the changes of these two phosphorylated proteins caused by DT-010 after 6 h of glutamate insult. As clearly demonstrated in **Figures 4C,D**, glutamate greatly decreased phosphorylation of GSK3β at Ser9 and Akt at Ser473 in CGNs. However, there was a approximate 40 and 60% increase in the protein levels of p-Akt and p-GSK3β in CGNs treated with DT-010, respectively, which was also significantly abrogated by LY.

### DT-010 Attenuates Glutamate-Evoked [Ca^2+^] Increase in CGNs

**FIGURE 5:**
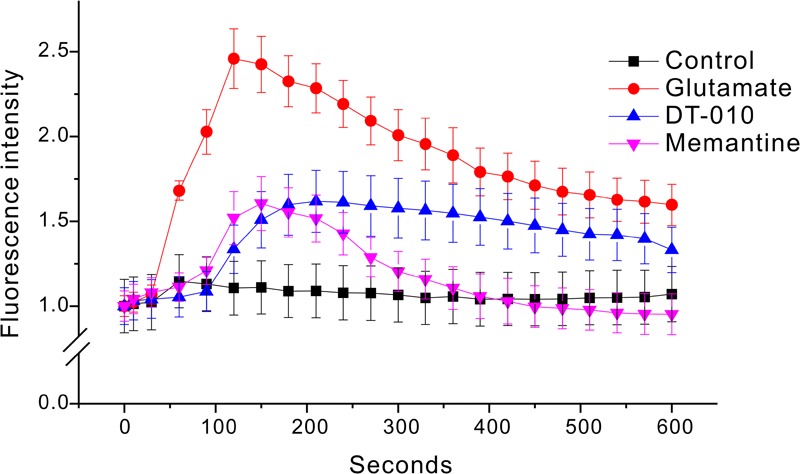
DT-010 markedly prevent the increase in [Ca^2+^] caused by glutamate in CGNs. CGNs were incubated with Fluo-4-AM (1 μM) for 30 min, and then washed twice to remove extracellular Fluo-4. CGNs were then pre-treated with DT-010 (30 μM) for 2 h, and then exposed to glutamate (100 μM). Time-dependent response of Ca^2+^ fluorescence intensity in each group was obtained using a confocal microscope.

Ca^2+^-dependence of toxic cell death is commonly observed after exposure of neurons to glutamate. To evaluate the Ca^2+^ homeostasis in CGNs treated by glutamate in the absence or presence of DT-010, Ca^2+^ imaging with Fluo-4 AM fluorescence was measured using confocal microscope. As shown in **Figure 5**, a persistent Ca^2+^ increase was observed for more than 10 min after exposure of CGNs to glutamate (100 μM). Two hour pre-treatment with DT-010 (30 μM) markedly blocked glutamate-induced Ca^2+^ rise.

### DT-010 Effectively Inhibits NMDA-Activated Whole-Cell Currents

**FIGURE 6:**
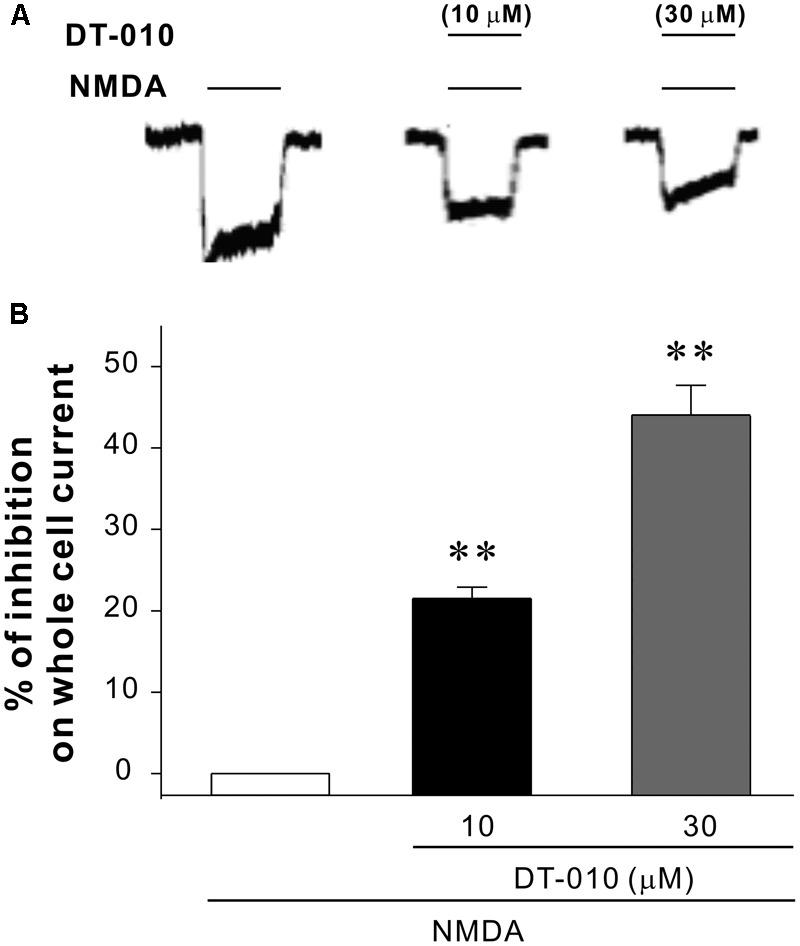
DT-010 inhibits NMDA-evoked whole cell currents in primary hippocampal neurons. **(A)** Representative current traces were taken from the same patch-clamped neuron showing DT-010 (10 and 30 μM) inhibition of NMDA-activated currents. **(B)** The statistical results of patch clamp analysis for the inhibition of DT-010 on NMDA-activated currents.

Ca^2+^ influx via the NMDA receptor channels plays a central role in glutamate excitotoxicity. Then to probe whether DT-010 could directly interact with NMDA receptors, high-fidelity analysis of underlying biophysical mechanisms were explored using the patch clamp technique in hippocampal neurons. It was evident in **Figure 6** that fast application of 30 μM NMDA elicited whole-cell currents. And as was expected, in the presence of DT-010 (10 and 30 μM), the NMDA receptor-mediated currents were significantly inhibited. Meanwhile, DT-010 itself did not affect the currents of hippocampal neurons (data not shown).

### DT010 Interacts with NMDA Receptor with the Binding Site Similar to MK-801

**FIGURE 7:**
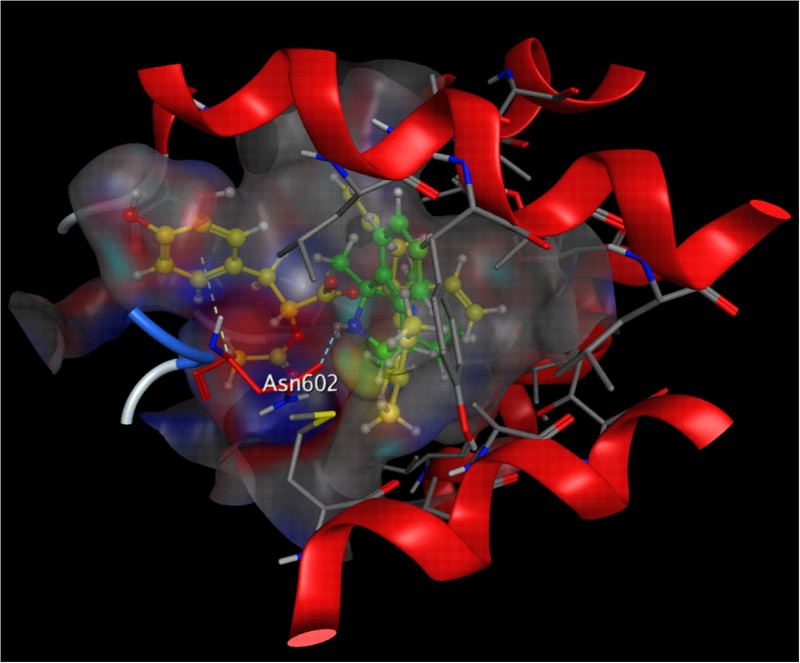
Molecular docking of DT010 and MK-801 on the NMDA receptor. A close-up view of the low energy pose of DT010 (yellow) and MK-801 (green) in the NMDA receptor channel pore.

To better investigate the acting site of DT-010 and the NMDA receptor, molecular docking simulation was performed. As shown in **Figure 7**, DT010 could interact with NMDA receptor at the ion channel, with free energy of binding of -8.7 kcal/mol. As a reference, the free energy of binding of MK-801 with NMDA receptor is -5.1 kcal/mol. Furthermore, DT010 (yellow) formed arene hydrogen bond to Asn602 of NMDA receptor, while MK-801 (green) formed NH group hydrogen bond to Asn602 of the receptor.

## Discussion

The progressive death of functional neurons following exposure to excessive glutamate causes progressive loss of learning function and underlies the pathology of AD. In this study, we identified DT-010, a dimeric derivative of DSS and TMP, as a novel NMDA receptor antagonist by whole-cell patching of hippocampal neurons, and systematically examined its protective effects against glutamate-mediated neurotoxicity in CGNs.

Dimerization, by linking one molecule to the same or another molecule, is believed to be one of the most effective and economical strategies to develop novel multifunctional compounds (Carlier et al., 2000; Terrillon and Bouvier, 2004). These generated homo- or hetero-dimers are most likely to possess pharmacological activities that their parent molecules lack. For instance, our group has previously developed several series of multifunctional dimers derived from tacrine and naturally occurring huperzine A (Carlier et al., 2000). Studies revealed that some of these anti-AD dimers exhibited differential anticholinergic-independent mechanisms including neuronal NOS inhibition (Li et al., 2007), NMDA receptor blockage (Luo et al., 2010), and/or neuronal differentiation-promoting properties (Hu et al., 2015a), which were not provided by either tacrine or huperzine A. In the present study, it was evident that our novel hetero-dimer DT-010 markedly protected against glutamate-induced excitotoxicity in purely cultured CGNs, an observation supported by the finding that DT-010 promoted neuronal viability and blocked the abnormal morphological changes. Under the same condition, its parent molecules, DSS and TMP, did not show any protective effects. These findings further support the proposition that dimerization strategy may produce multifunctional molecules with novel functional features and thereby represent an efficient approach for drug development.

Why did DT-010 exert such neuroprotection against glutamate-induced excitotoxicity? To uncover the mechanism of neuroprotective action, we systematically investigated whether and how DT-010 affected several key targets/steps of glutamate insult, from the downstream signaling cascade (PI3-K/Akt/GSK3β pathway) to Ca^2+^ influx and to the upstream NMDA receptors.

First, we examined the effects of DT-010 on GSK3β, as GSK3β is a common but vital downstream signaling pathway of glutamate insult. GSK3β is activated on two phosphorylation sites (Tyr-216 and Ser-9), which have opposite effects. Phosphorylation on Tyr-216 results in GSK3β activation while Ser-9 phosphorylation attenuates its activity, indicating that GSK3β is regulated negatively by the phosphorylation of serine 9. Enhanced GSK3β levels were found in a variety of *in vitro* and *in vivo* models associated with AD (Barbero-Camps et al., 2013; Llorens-Martin et al., 2014), suggesting GSK3β contributes significantly to the neuronal degeneration. PI3-K/Akt is one of the most important upstream signaling components that regulate GSK3β phosphorylation at ser9. Our findings that DT-010 reversed the glutamate-induced decrease in GSK3β at Ser9 site, and that LY294002 abrogated the neuroprotective effects of DT-010 indicated that this identified molecule provided its neuroprotection through the inhibition of GSK3β in a PI3-K/Akt-dependent pathway. The direct role of DT-010 on GSK3β activity will be further revealed in our future projects by enzymatic activity assay and molecular docking analysis.

Ionotropic and metabotropic glutamate receptors are synaptic receptors located on the post-synaptic neurons. NMDA receptor is one of the three types of ionotropic receptors, the others being the α-amino-3-hydroxy-5-methyl-4-isoxazole propionic acid (AMPA) and kainate receptors. It has been reported that NMDA receptor has the highest affinity for the ligand (glutamate), and that NMDA receptor acts as the primary target in response to glutamate excitotoxic insult. Indeed, we have previously demonstrated that blockage of NMDA receptors by memantine and MK801, two selective NMDA receptors, greatly prevented glutamate-induced neurotoxicity including neuronal death, accumulation of ROS, Ca^2+^ influx and inhibition of pro-survival PI3-K/Akt/GSK3β pathway (Pi et al., 2004; Li et al., 2007; Hu et al., 2013). Furthermore, transfection of NMDA receptor subunits into cells in the presence of glutamate-containing culture medium resulted in neuronal death that could be attenuated by NMDA receptor antagonists (Cik et al., 1993; Anegawa et al., 1995). All the findings suggest that excitotoxicity associated with aberrant glutamate signaling is attributable largely to NMDA receptors. Therefore, in the current study, we investigated the neuroprotection of DT-010 against excitotoxicity caused by glutamate, with a special emphasis on the NMDA receptor inhibition. The effects of DT-010 on APMA and kainate receptors will be systematically examined in our future projects.

Considerable evidence has amassed that pathological activation of NMDA receptors, either by direct or indirect mechanisms, results in excessive Ca^2+^ influx into the cytoplasm, which can lead to cell death (Li et al., 2007; Dar et al., 2017). Further, based on the fact that the pro-survival PI3-K/Akt/GSK3β acts a downstream signaling pathway of Ca^2+^, and that DT-010 prevented the glutamate-induced Ca^2+^ influx in CGNs as assayed by Fluo-4 AM fluorescence, it is reasonably to ask whether DT-010 reversed the increase in Ca^2+^ concentration directly by blocking NMDA receptors. As was excepted, DT-010 effectively inhibited NMDA-activated whole-cell currents in cultured hippocampal neurons, strongly suggesting the neuroprotection of DT-010 was attributable to a direct effect on NMDA receptor channels. Furthermore, to pecifically clarify the involvement of NMDA receptor in DT-010-mediated neuroprotection, we introduced a cell model in which neurotoxicity was induced in CGNs by NMDA, an inducer that specifically over-activate NMDA receptors. DT-010, together with other NMDA receptor antagonists (memantine and MK801), greatly prevented NMDA-induced neurotoxicity (data not shown). At last, molecular docking simulation analysis further revealed a possible binding mode that inhibited NMDA receptor at the ion channel, showing that DT-010 favorably binds to Asn602 of NMDA receptor via arene hydrogen bond. All the results, taken together, strongly indicated that DT-010 protected neurons against glutamate-induced excitotoxicity through the blockage of NMDA receptors.

It has been well documented that synaptic and extrasynaptic NMDA receptors have distinct compositions and couple with opposing roles. Once activation, synaptic NMDA receptors usually initiate physiological changes for the induction of synaptic plasticity and tend to promote neuronal survival, while extrasynaptic NMDA receptors are associated with excitotoxic pathological conditions and often cause neurodegeneration observed in AD (Kaufman et al., 2012; Karpova et al., 2013; Lesept et al., 2016; Sun et al., 2016). Since there is evidence linking extra-synaptic NMDA receptor activation to glutamate-induced excitotoxicity (Chen and Diamond, 2002; Talantova et al., 2013), we speculate that DT-010 may provide its neuroprotection via inhibiting extrasynaptic NMDA receptors.

**FIGURE 8:**
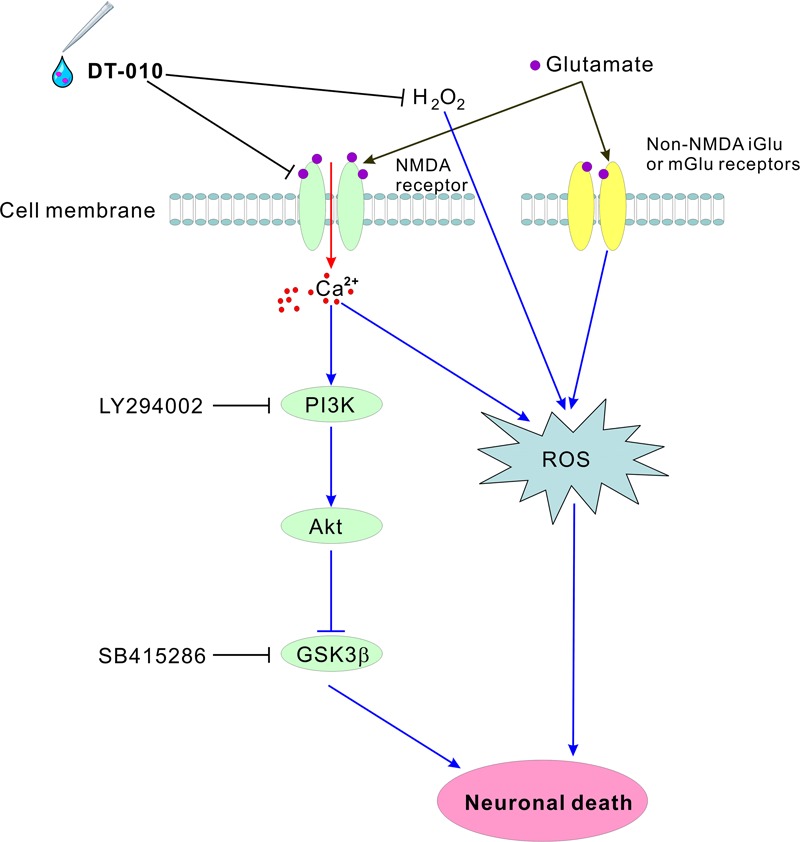
Schematic diagram shows the possible molecular pathways mediated in the neuroprotective effects by DT-010. DT-010 antagonizes NMDA receptor at the synapse of cell membrane, subsequently blocks the intracellular Ca^2+^ influx, activates pro-survival PI3-K/Akt pathway to inhibit GSK3β, and finally protects CGNs from glutamate-induced neurotoxicity.

In addition to NMDA receptors, excitotoxic glutamate could also over-activate metabotropic glutamate receptors at most excitatory synapses, and subsequently leads to oxidative stress. This partially explained why DT-010 almost completely blocked endogenous accumulation of ROS caused by glutamate and exogenous oxidative stimulus, while not fully protected neurons from glutamate-induced cytotoxicity (**Figure 8**). In other words, we could not exclude any other possible contributing factors that DT-010 may target to provide neuroprotection in the cell membrane or cytoplasm.

Accumulating lines of evidence indicate that multifactorial etiopathogenesis underlie the pathogenesis of AD, and that this devastating disease requires multi-factorial intervention to target different pathological sites (Iqbal and Grundke-Iqbal, 2010). Molecules with multifunctional properties might offer stronger therapeutic efficacy to combat AD by addressing various pathological sites synergistically. The stimulation of myocyte enhancer factor 2 proven previously (Hu et al., 2016), NMDA receptor blockage, anti-oxidant and neuroprotective effects demonstrated herein may provide DT-010 as a potent drug candidate for treating neurodegenerative diseases, AD in particular.

In summary, our present study provided clear evidence that DT-010, the derivative of TMP and DSS, offered great neuroprotection against glutamate-induced excitotoxicity in primary neurons, by blocking key steps of glutamate insult, from the upstream NMDA receptors to Ca^2+^ influx and to downstream GSK3β cascade. DT-010 could be served as a novel NMDA receptor antagonist.

## Author Contributions

HL, ZZ, and YH designed the overall project. SH directed experiments in various biochemical analyses including preparation of primary neurons and assays of MTT, FDA/PI staining, and Western blot, and drafted the manuscript. HH and LS performed the DPPH radical scavenging assay. SM performed the Ca^2+^ imaging with Fluo-4 fluorescence assay and physiological experiments. GC carried out the molecular docking analysis. HL, ML, ZZ, and YH revised the manuscript. All authors read and approved the final manuscript.

## Conflict of Interest Statement

The authors declare that the research was conducted in the absence of any commercial or financial relationships that could be construed as a potential conflict of interest.

## Abbreviations

AD: Alzheimer’s disease
CGNs: cerebellar granule neurons
DCFH-DA: 2′7′-dichlorodihydrofluorescein diacetate
DIV: days *in vitro*
DSS: danshensu
FDA: fluorescein diacetate
GSK3β: glycogen synthase kinase 3beta
MTT: 3(4,5-dimethylthiazol-2-yl)-2.5-diphenyltetrazolium bromide
NMDA: *N*-Methyl-D-aspartate
PI: propidium iodide
PI3-K: phosphoinositide 3-kinase
ROS: reactive oxygen species
SD: Sprague-Dawley
TMP: tetramethylpyrazine
